# Determinants of Acute Kidney Injury in Children Undergoing Cardiopulmonary Bypass: Single-Center Experience in Saudi Arabia

**DOI:** 10.7759/cureus.32666

**Published:** 2022-12-18

**Authors:** Abdullah Alzahrani, Raghad A Alahmadi, Sara K Alghamdi, Rahaf A AlQurashi, Mohammed Y Al-Hindi

**Affiliations:** 1 Pediatrics, King Abdulaziz Medical City, Ministry of National Guard Health Affairs, Jeddah, SAU; 2 College of Medicine, King Saud Bin Abdulaziz University for Health Sciences, Jeddah, SAU; 3 Research and Development, King Abdullah International Medical Research Center, Jeddah, SAU; 4 College of Medicine, King Saud Bin Abdulaziz University for Health Sciences, jeddah, SAU

**Keywords:** renal function, creatinine, cardiac surgery, acute kidney injury, cardiopulmonary bypass

## Abstract

Introduction

Cardiopulmonary bypass (CPB) is a machine used in open cardiac surgeries and has been linked to many complications, one of which is acute kidney injury (AKI). Also, the Kidney Disease Improving Global Outcomes (KDIGO) criteria are used to diagnose AKI in the pediatric population. The study aimed to investigate the association between cardiopulmonary bypass duration and renal function impairment in pediatric patients who had cardiac surgery.

Methods

This was an observational, cross-sectional study conducted at the King Abdulaziz Medical City, King Faisal Cardiac Center, the section of the Pediatric Cardiac Intensive Care Unit (PICU), Ministry of National Guard Health Affairs, from January 2016 to December 2019.

Patients younger than 14 years old, those having a cardiac surgery where CPB was implemented, normal pre-operative kidney functions, and having a cardiac surgery longer than 60 minutes (min) were included. The exclusion criteria were patients known to have pre-operative renal impairment and patients with pre-operative hemodynamic instability or cardiac arrest.

Demographics of pre-operative, intra-operative, and post-operative data were extracted, and Statistical Package for the Social Sciences (SPSS) version 25 (Armonk, NY: IBM Corp.) was used for analysis. For descriptive statistics, frequencies and percentages for qualitative data were examined, while mean and standard deviation (SD) or median and interquartile range (IQR) quantitative data were used accordingly. Student's t-test, Mann-Whitney (median test), chi-square, or Fisher's exact tests were used for univariate analysis accordingly. Logistic regression analysis was used to determine significant predictors for developing AKI. A p-value of <0.05 would be considered significant.

Results

Of the 111 patients, 87 patients were included in the analysis. The median age was six months, IQR two to 13 months, body mass index (BMI) mean of 13.8, and SD 3.6. There was similar sex distribution, male 47.1% vs. female 52.9%. There were no patients in Risk Adjustment for Congenital Heart Surgery (RACHS) who scored 5 or 6. The AKI prevalence was 31% (27/87) within three days after surgery. One patient had stage 2 AKI; the rest were mild. One patient (3.7%) died. The CPB time was significantly longer in patients who developed AKI 150 (104-202), vs. non-AKI 104 (82-142) min, p=0.004. In the AKI group, the mean baseline (pre-operative) serum creatinine (sCr) was significantly lower, whereas, it was significantly higher at 24 hours (h), and 48 h post-operation (p=0.001, 0.001, and 0.036, respectively). Additionally, the estimated Glomerular Filtration Rate (eGFR) was significantly higher in the AKI group at 24 h (p=0.007).

In logistical regression analysis, CPB time (per min unit time) was a significant predictor for developing AKI, OR 1.015, p=0.011 as a measured outcome. However, only CPB time >180 min was highly significant with OR 16.2, p=00.6 compared to CPB time 121-180 min OR 2.3, p=0.29 and CPB time 91-120 min OR 1.2, p=0.84.

Conclusion

Acute kidney injury is an expected complication of pediatric congenital heart surgery receiving CPB. Although in our single-center experience, CPB duration was a significant predictor for AKI; however, it is considered a mild complication that does not contribute significantly to short-term morbidity or mortality. A larger multicenter, national prospective data registry is recommended to explore long-term effects.

## Introduction

Cardiopulmonary bypass is considered a sophisticated procedure that has become standard care in open cardiac surgeries, especially in infants and young children with congenital heart defects. Cardiopulmonary bypass enables surgeons to operate on the heart without interfering with its role. Cardiopulmonary bypass oxygenates and removes carbon dioxide from the blood, then returns it to the body through an arterial cannula with an adequate flow to maintain circulation during the surgery and provide the surgeon with a reasonably dry, bloodless surgical field [1,2].

Cardiopulmonary bypass might lead to various complications, such as renal function impairment [3], hepatic impairment [4], hyperglycemia, and systemic inflammatory response syndrome [5]. One of the most common renal function impairments that might develop is AKI, a rising complication in the pediatric population. Acute kidney injury (previously called acute renal failure) is a sudden kidney failure or kidney damage episode that happens within a few hours or days. It causes a build-up of waste products in the blood and makes it hard for the kidneys to keep the right balance of fluid in the body [6]. The incidence of AKI among pediatric patients that are between 31 days and 21 years of age who were admitted to the PICU in a single US center is 11% [7]. Several diagnostic criteria detect acute kidney injury. The most recent and modified one is the Kidney Disease Improving Global Outcomes (KDIGO) criteria which categorize the patients based on the changes in the sCr levels or urine output. In addition, AKI can be diagnosed by other biomarkers, such as eGFR and blood urea nitrogen (BUN) [8].

Few published literature have shown an association between cardiopulmonary bypass duration and renal function impairment in the pediatric population. Li et al. report an AKI incidence of 42% based on the Acute Kidney Injury Network classification system [9]. In Saudi Arabia, no published data measures the prevalence and associated risk factors of AKI post-cardiopulmonary bypass (CPB) use based on the latest KDIGO criteria. Therefore, the study aimed to measure the prevalence of AKI in pediatrics who underwent cardiac surgery for congenital heart disease requiring CPB using the KDIGO criteria, and to explore additional risk factors associated with the development of AKI, such as intensive care unit (ICU) length of stay and mechanical ventilation, and to measure the association between CPB duration and different renal function profile and morbidities.

## Materials and methods

Study design and area and settings

This was an observational, cross-sectional study conducted at King Abdulaziz Medical City, King Faisal Cardiac Center, section of the Pediatric Cardiac Intensive Care Unit, Ministry of National Guard Health Affairs, from January 2016 to December 2019. Institutional Board Review Office at King Abdullah International Research Office approved the study with reference number IRBC/0883/19.

Identification of study participants

The inclusion criteria include patients younger than 14 years old, having cardiac surgery where CPB was implemented, normal pre-op kidney functions, and having cardiac surgery lasting more than 60 min. The exclusion criteria were patients known to have pre-op renal impairment and patients with pre-operative hemodynamic instability or cardiac arrest.

Sampling technique and data collection process

Non-probability, consecutive sampling technique was used to select eligible patients. The electronic health records were screened for children admitted to the PICU who required cardiac surgery with CPB between January 2016 to December 2019. Demographics of pre-operative and post-operative data were extracted, and the inclusion and exclusion criteria were applied to find our representative sample. The variable extracted included the patient's demographics, e.g., age, weight, and height. Also, it had pre-operative, intra-operative, and post-operative characteristics and morbidities like fluid balance, the use of nephrotoxic medications, the use of angiogram, cross-clamping time, intraoperative bypass duration, use of peritoneal dialysis (PD) catheter, estimated GFR, ventilation duration, ionotropic score, renal angina index (RAI), extracorporeal membrane oxygenation (ECMO), hemoglobin, BUN, sCr levels, the central venous pressure, urine output, blood transfusion, ICU length of stay, and ventilator-free days in the ICU. Also, mortality was recorded and examined for its relation to the procedure.

Case definition: KDIGO is defined by the increase in sCr by ≥0.3 mg/dL (≥ 26.5 μmol/L) within 48 h or an increase in sCr to ≥1.5 times baseline which is known or presumed to have occurred within the previous 7 days, or urine volume <0.5 mL/kg/h for 6 h [8]. Further definitions for the severity of AKI are shown in Appendix 1, and other variable definitions are in Appendix 2.

Data analysis

An online statistical software, OpenEpi [10], was used to calculate the sample size based on a previous study [9] that estimated a difference of 30% of patients who are exposed to pronged CPB time and developed AKI (51%) compared to unexposed (21%). The ratio of unexposed/exposed is 2:1, the calculated sample size in exposed is 29, the sample size unexposed is 57, and the total is 86. Such a sample is needed to detect a statistical significance difference assuming a significance level of 95% and a power (1-beta) of 80%,

We used Microsoft Excel files to manage the data, and we will then transfer them to Statistical Package for the Social Sciences (SPSS) version 25 for analysis. For descriptive statistics, frequencies and percentages for qualitative data were examined. At the same time, mean and standard deviation (SD) or median and interquartile range (IQR) will be produced for quantitative data in normally distributed or skewed data, respectively. Student t-test was used for comparing two groups for normally distributed data, and the Mann-Whitney test (median test) for skewed data. Chi-square or Fisher exact test was used to comparing between categorical data. Logistic regression analysis was used to determine significant predictors for developing AKI. Collinearity was assessed, and those with high correlation (r>0.45) were eliminated from the model. The model adjusted for age, gender, and body mass index (BMI). It included CBP time, Risk Adjustment in Congenital Heart Surgery (RACHS) score (score of 2 as reference), RAI (score of 5 as reference), and baseline creatinine as independent variables. A P-value of <0.05 would be considered significant.

## Results

We studied 111 children who underwent heart surgery for congenital defects between January 1, 2016, and December 31, 2019. Of the 111 patients, 14 were excluded from the analysis because their cardiac surgery duration was less than 60 min or they were hemodynamically unstable and known to have pre-operative renal impairment. Another 10 patients were excluded because they had missing data or incomplete medical records. Hence 87 patients were included in the analysis. The median age was six months, IQR two to 13 months, BMI mean of 13.8, and SD 3.6. There was similar sex distribution, male 47.1% vs. female 52.9%. There were no patients in RACHS who scored 5 or 6.

**Figure 1:**
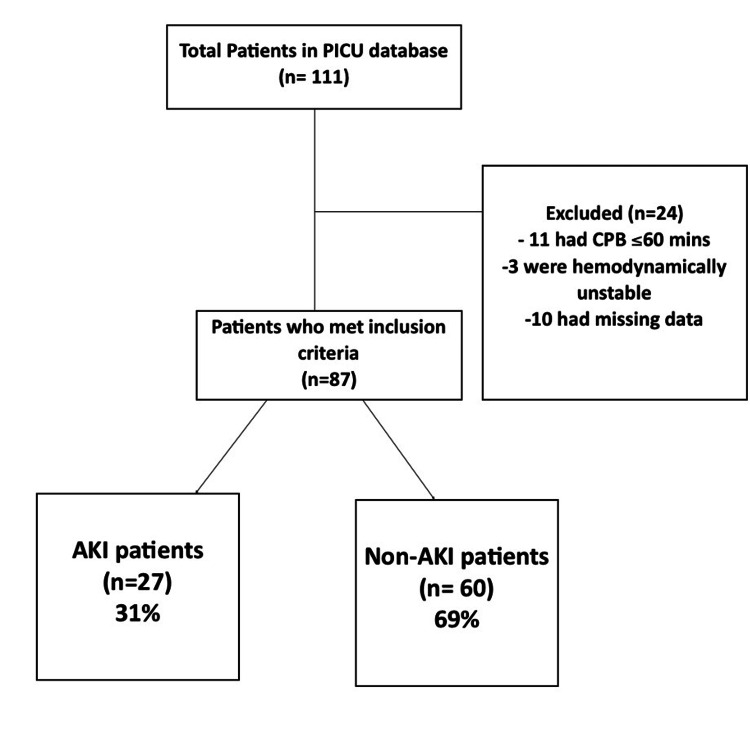
Flow chart for patient recruitment. PICU: pediatrics intensive care unit; CPB: cardiopulmonary bypass; AKI: acute kidney injury

Furthermore, we screened for acute kidney injury according to the KDIGO criteria (case definition of AKI in Appendix 1) and ended up with 27 patients who met the criteria-making (further details are shown in Appendix 3); the AKI prevalence in our population of 31% within three days after surgery (Figure 1).

**Figure 2:**
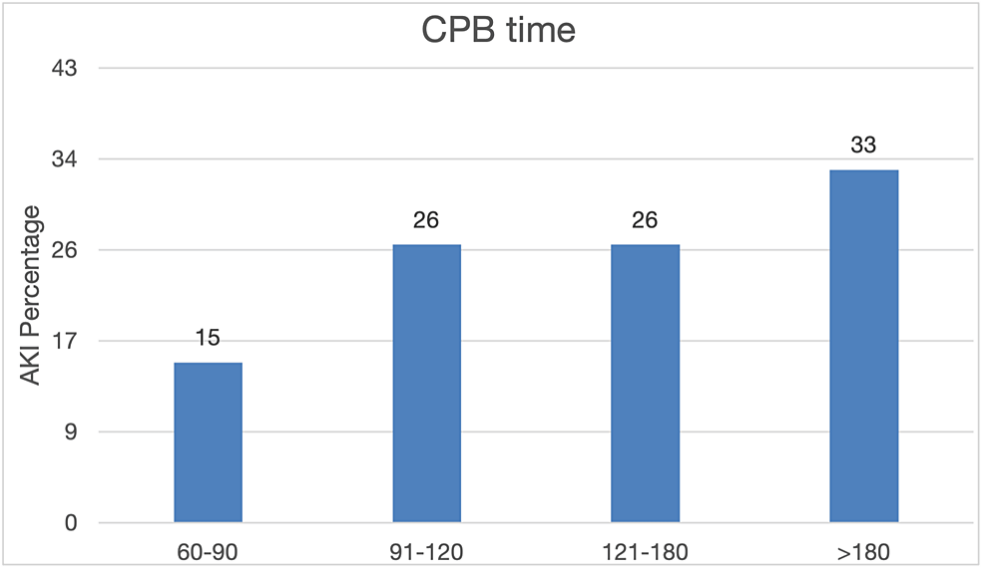
AKI prevalence and CPB time. CPB: cardiopulmonary bypass; AKI: acute kidney injury

There was a stepwise increase of AKI prevalence with the rise of CPB time, where four patients (15%) had a CPB time of 61-90 min, seven (26%) had 91-120 min, seven (26%) had 121-180 min, and nine patients (33%) had a CPB time of equal to or more than 180, Fisher's exact p=0.007, and Spearman correlation 0.34, p=0.001 (Figure 2). Interestingly only one patient had a moderate case of AKI, stage 2; the rest were mild. In addition, one out of the 27 patients was a death case, making our cohort's mortality rate (3.7%).

The baseline characteristics of our cohort are shown in Table 1. The only significant variable was the baseline creatinine (p=0.001). Other variables such as comorbidities, RACHS score, renal angina index (see Appendix 3 for definitions), pre-operative nephrotoxic medications, pre-operative PD catheter, and pre-operative cardiac angiography were insignificant.

**Table 1:** Baseline characteristics of study patients. *Chi-square test. **Mann-Whitney test. ***Fisher's exact test. For definitions of variables, please refer to Appendix 2.

Baseline characteristics		Non-AKI	AKI	p-Value
Gender	Female	29 (48.3%)	12 (44.4%)	0.737*
	Male	31 (51.7%)	15 (55.6%)	
Age (months), median (IQR)		7 (3-14)	5 (1-7)	0.119**
Body mass index, mean (standard deviation)		13.83 (±4.12) Std. error (0.53)	13.88 (±2.21) Std. error (0.425)	0.951**
Baseline creatinine (umol/L)		39 (35-42)	36 (33-38)	0.001**
RACHS score	2	32 (53.3%)	8 (29.6%)	0.121*
	3	18 (30%)	12 (44%)	
	4	10 (16.7%)	7 (25.9%)	
Comorbidities	No	33 (55%)	15 (55.6%)	0.962*
	Yes	27 (45%)	12 (44.4%)	
Pre-operative nephrotoxic medications	No	51 (85%)	20 (74.1%)	0.224*
	Yes	9 (15%)	7 (25.9%)	
Pre-operative prophylactic PD catheter	No	47 (78.3%)	21 (77.8%)	0.954*
	Yes	13 (21.7%)	6 (22.2%)	
Pre-operative cardiac angiography	No	55 (91.7%)	27 (100%)	0.122*
	Yes	5 (8.3%)	0 (0%)	
Renal angina index	5	39 (65%)	15 (55.6%)	0.525***
	10	15 (25%)	7 (25.9%)	
	20	4 (6.7%)	2 (7.4%)	
	40	2 (3.3%)	3 (11.1%)	
Blood transfusion during the operation	No	6 (10%)	2 (7.4%)	1***
	Yes	54 (90%)	25 (92.6%)	

The CPB time was significantly longer in patients who developed AKI 150 (104-202) min, vs. non-AKI 104 (82-142) min, p=0.004. Secondary outcomes included sCr measures at baseline (pre-operative), immediately post-operative then every 24 h up to three days after surgery. In the AKI group, the mean baseline sCr was significantly lower, whereas, it was significantly higher at 24 h, and 48 h post-operation (p=0.001, 0.001, and 0.036, respectively). Additionally, the estimated GFR in the AKI group was significant at 24 h (p=0.007) compared to the non-AKI group. Other outcomes, such as inotropic score, blood urea nitrogen, ventilation duration, and ICU length days, were not statically significant; details are shown in Table 2.

**Table 2:** Intra-operative and post-operative outcomes. *Chi-square test. **Mann-Whitney test. ***Fisher's exact test. For definitions of variables, please refer to Appendix 2.

Outcome		Non-AKI	AKI	p-Value
Cardiopulmonary bypass time (min)		104.5 (83.25-142.5)	150 (104-202)	0.004**
Cross clamp time (min)		66 (53-83)	73 (48-102)	0.640**
ECMO	No	59 (98.3%)	24 (88.9%)	0.087***
	Yes	1 (1.7%)	3 (11.1%)	
Renal angina index	5	39 (65%)	15 (55.6%)	0.525***
	10	15 (25%)	7 (25.9%)	
	20	4 (6.7%)	2 (7.4%)	
	40	2 (3.3%)	3 (11.1%)	
Ventilation duration		2 (1-5)	3 (1-8)	0.272**
Injury	1	38 (63.3%)	15 (55.6%)	0.559***
	2	16 (26.7%)	7 (25.9%)	
	4	4 (6.7%)	2 (7.4%)	
	8	2 (3.3%)	3 (11.1%)	
Risk	5	60 (100%)	27 (100%)	-
Fluid overload		-0.77 (±9.53)	-0.97 (±14.09)	0.94**
Inotropic score	Inotropic score-0	9 (7-13)	12 (7-18)	0.432**
	Inotropic score-24	7 (5-10)	9 (5-15)	0.110**
	Inotropic score-48	5 (0.5-7.75)	5 (2.5-10)	0.245**
	Inotropic score-72	3 (0-6)	5 (0-9)	0.146**
Creatinine	Baseline (pre-operative) creatinine	39 (35-42)	36 (33-38)	0.001**
	Creatinine-0	50 (45-54)	52 (49-57)	0.119**
	Creatinine-24	44 (38-51)	52 (43-64)	0.001**
	Creatinine-48	40 (35-45)	42 (38-57)	0.036**
	Creatinine-72	38 (33-42)	39 (36-46)	0.223**
eGFR	eGFR-0	53 (45-68)	50 (38-60)	0.315**
	eGFR-24	62 (44-80)	48 (39-58)	0.007**
	eGFR-48	69 (49-89)	57 (44-73)	0.053**
	eGFR-72	70 (53-92)	61 (46-80)	0.107**
Urine output	Urine output-0 (mL/kg/h)	2 (1.5-3.8)	2 (1.3-4.5)	0.819**
	Urine output-24 (mL/kg/h)	3 (2.1-4.2)	2 (1.9-3.9)	0.094**
	Urine output-48 (mL/kg/h)	3 (2.6-5.1)	4 (2.9-5.2)	0.938**
	Urine output-72 (mL/kg/h)	4 (2.8-5.7)	3 (2.9-5.5)	0.544**
Fluid balance	Fluid balance-0	88 (-17-235)	125 (-65-383)	0.808**
	Fluid balance-24	28 (-72-310)	48 (-70-227)	0.901**
	Fluid balance-48	-83 (-194-98)	-102 (-220-7.5)	0.320**
	Fluid balance-72	-108 (-243-28)	-110 (-234-12.7)	0.710**
Hemoglobin (g/dL)		11.03 (±1.51)	11.80 (±2.17)	0.060**
BUN (mmol/L)		4.72 (±1.59)	5.51 (±2.10)	0.055**
ICU length days		7 (4-12)	7 (5-17)	0.190**

In logistical regression analysis, CPB time (per min unit time) was a significant predictor for developing AKI, beta coefficient 0.015, Wald 6.5, OR 1.015, p=0.011 as a measured outcome. However, if we replace the model with the CPB time as 30 min unit time where CPB time 30-60 min is the reference, then there is a stepwise increase odd fo developing with each unit of increase in CPB time; however, only CPB time >180 min was highly significant with OR 16.2, p=00.6 compared to CPB time 121-180 min OR 2.3, p=0.29 and CPB time 91-120 min OR 1.2, p=0.84. Another independent predictor was baseline sCr with a beta coefficient of -0.14, Wald 5.4 OR=0.87, and p=0.02.

## Discussion

This single tertiary center study showed an association between cardiopulmonary bypass duration and the deterioration of renal function in children post-cardiac surgery. The prevalence of AKI (31%) according to the KDIGO criteria. Our prevalence is lower than previously reported by Li et al. and Aydin et al., who had 42% and 51%, respectively [9,11]. Our sample's characteristics were comparable with the multicenter study of Li et al. apart from the smaller age group; however, their sample was collected more than a decade ago. Their study established a benchmark for AKI prevalence in children post-cardiac surgery for congenital heart disease [9].

Cardiopulmonary bypass is the mainstay management in open heart surgeries as a replacement for the heart, oxygenating and removing carbon dioxide from the blood, then returning it to the body through an arterial cannula with adequate flow to maintain the circulation during the time of the surgery [2]. Although the pathophysiology is still unclear, CPB was linked to renal vasoconstriction and redirection of the blood away from the kidney, which, if merged with hemodilution, decreases renal oxygen delivery by 20% during the surgery. On the other hand, renal demand for oxygen is unchanged. This mismatch in renal oxygenation is accompanied by a release of a tubular injury marker that further aggravates renal functions after weaning from CPB [3].

Many studies have examined that the associated time of the CPB may be considered a risk factor for developing kidney insult in adults [12] and only a few children; our study adds to the body of literature this association in young children [13]. Moreover, there was a stepwise increase in AKI with each 30 min increase in CPB time beyond 60 min. The most pronounced was CPB time of >180 min, with 16 times the odds of developing AKI even after adjusting for modifiable factors. This association was consistent with the literature, which found a stepwise increasing risk for developing AKI with longer durations of CPB, with the highest incidence at >180 min [9].

We explored the association between the Renal Angina Index score and AKI, which is used as a predictive tool to calculate the risk of developing AKI upon ICU admission, results in univariate and regression analysis didn't find an association. To our knowledge, we didn't see a study that examined such an association. Pre-operative baseline creatinine was lower in the group of patients who developed AKI. However, this was most likely due to expected age-associated lower creatinine values, which is evidenced by the fact that the baseline estimated glomerular filtration rates were not statistically different between the AKI and non-AKI groups [9]. In our study, only two patients had been put on ECMO, and only one died in the AKI group [14]. Such severe complications and mortality are consistent with the study by Li et al. (1.9%) and Aydin et al. (7.3%) [9,11].

Our study is the first to analyze the association between CBP time and AKI in children undergoing heart surgery for congenital heart disease in Saudi Arabia. It also was the first to examine the association between renal angina score and AKI. On the other hand, our study did not include patients with RACHS-1 because they were all simple procedures and ended up with a CPB time of less than 60 min [15]. In addition, our study was limited by being conducted in one center compared to their multicenter cohort by Li et al., they included patients up to 17 years of age, also included patients who had a duration of CPB less than 60 min, while in our cohort we had to exclude anyone older than 14 years old. However, our results did not differ [9].

We recommend having a larger national multicenter prospective study to elaborate more on further modifiable risk factors in our population such as maternal antenatal risk [16] and multidomain socioeconomic risk score [17]. Long-term follow-up is also recommended to explore the effect on neurodevelopment [18,19], healthcare utilization, growth, renal function, and possibly adulthood complications [20]. Further studies are needed for the renal function analysis to clarify the long-term follow-up.

## Conclusions

 Acute kidney injury is an expected complication of pediatric congenital heart surgery receiving CPB. Although in our single-center experience, CPB duration was a significant predictor for AKI; however, it is considered a mild complication that does not contribute significantly to short-term morbidity or mortality. a larger multicenter, national prospective data registry is recommended to explore long-term effects.

## Appendices

Appendix 1 

**Table 3:** Staging for AKI as per KDIGO. AKI: acute kidney injury; KDIGO: Kidney Disease Improving Global Outcomes

AKI Stage	Serum creatinine (sCr)	Urine output
Stage 1	1.5-1.9 times baseline OR ≥26.5 μmol/L increase	<0.5 mL/kg/h for 6-12 h
Stage 2	2.0-2.9 times baseline	<0.5 mL/kg/h for ≥12 h
Stage 3	3.0 times baseline OR sCr ≥353.6 μmol/L OR initiation of renal replacement therapy OR, eGFR <35 mL/min per 1.73 m^2^	<0.3 mL/kg/h for ≥24 h OR anuria for ≥12 h

Appendix 2 

**Table 4:** Definitions of terminology and abbreviations used. RACHS: Risk Adjustment for Congenital Heart Surgery; CPB: cardiopulmonary bypass; eGFR: estimated Glomerular Filtration Rate

Terminology/ abbreviation	Definition
CPB	A Cardiopulmonary bypass machine replaces the heart, oxygenating and removing carbon dioxide from the blood, then returning it to the body through an arterial cannula with adequate flow to maintain circulation during the surgery
Aortic cross-clamp	A surgical instrument used in cardiac surgery to clamp the aorta and separate the systemic circulation from the heart's outflow
Ventilation duration	Days that were spent on mechanical ventilation.
Inotropic score-0	The amount of cardiovascular support required by infants post-operatively includes dopamine, dobutamine, epinephrine, milrinone, vasopressin, and norepinephrine after the surgery on day 1
Inotropic score-24	The amount of cardiovascular support required by infants post-operatively includes dopamine, dobutamine, epinephrine, milrinone, vasopressin, and norepinephrine 24 h after surgery
Inotropic score-48	The amount of cardiovascular support required by infants post-operatively includes dopamine, dobutamine, epinephrine, milrinone, vasopressin, and norepinephrine 48 h after surgery
Inotropic score-72	The amount of cardiovascular support required by infants post-operatively includes dopamine, dobutamine, epinephrine, milrinone, vasopressin, and norepinephrine at 72 h after surgery
RACHS score	The Children's Hospital Boston team developed it through a panel of 11 nationally representative pediatric cardiologists and cardiac surgeon members. Initially using clinical judgment, with further refinement based on two national databases' data, it allocated 207 surgical procedures in 6 different categories with similar risks for hospital mortality. Three additional clinical factors (age, prematurity, and noncardiac congenital structural abnormalities)
RACHS score-1	Patent ductus arteriosus (PDA) >30 d, ostium Secundum atrial septal defect (OS ASD), sinus venosus septal defect, aortic coarctation>30d, partial anomalous pulmonary venous connection (PAPVC)
RACHS score-2	Ventricular septal defect (VSD), tetralogy of Fallot (TOF), Glenn, ostium primum atrial septal defect (OP ASD), aortic coarctation at age <30 d, ASD and VSD, repair of total anomalous pulmonary veins at age >30 d
RACHS score-3	Fontan procedure, systemic to pulmonary artery shunt, mitral valvotomy or valvuloplasty, mitral valve replacement (MVR), pulmonary artery (PA) banding
RACHS score-4	Arterial switch operation with VSD closure, atrial septectomy, and total anomalous pulmonary veins repair at age <30 d
Renal angina index	Determined based on changes in the creatinine in predicting persistent AKI in adult patients admitted from general wards
ECMO	Extracorporeal membrane oxygenation is used when the patient's lungs fail to support their breathing or the heart fails to pump oxygen
Renal replacement therapy	Therapy that replaces the normal blood-filtering function of the kidneys. It is used when the kidneys are not working well, which is called kidney failure and includes acute kidney injury and chronic kidney disease
Creatinine-0	Creatinine is a waste product produced by muscles from the breakdown of a compound called creatine, measured on day 1 after surgery
Creatinine-24	Measurement of creatinine 24 h after surgery
Creatinine-48	Measurement of creatinine at 48 h after surgery
Creatinine-72	Measurement of creatinine at 72 h after surgery
eGFR-0	The estimated glomerular filtration rate is a test to measure the level of kidney function and determine the stage of kidney disease measured on day 1 after surgery
eGFR-24	The estimated glomerular filtration rate measured 24 h after surgery
eGFR-48	Estimated glomerular filtration rate measured at 48 h after surgery
eGFR-72	Estimated glomerular filtration rate measured at 72 h after surgery
Urine output-0 (mL/kg/h)	Normal urine output of 0.5 to 1.5 mL/kg/h, measured on day 1 after surgery
Urine output-24 (mL/kg/h)	Urine output measured 24 h after surgery
Urine output-48 (mL/kg/h)	Urine output measured 48 h after surgery
Urine output-72 (mL/kg/h)	Urine output measured at 72 h after surgery
Fluid balance-0	Total fluid minus the output measured on day 1 after surgery
Fluid balance-24	Total fluid minus the output measured 24 h after surgery
Fluid balance-48	Total fluid intake minus the output measured at 48 h after surgery
Fluid balance-72	Total fluid intake minus the output measured at 72 h after surgery
Co-morbidity	Any congenital disorder that the patient has other than their cardiac defect (including Down syndrome, failure to thrive, sickle cell anemia)
Pre-op nephrotoxic medication	Use of nephrotoxic medications such as gentamicin, vancomycin, and amoxicillin
ICU length days	Days that were spent in the pediatric intensive care unit
Ventilator-free days in ICU	Days that were spent in the intensive care unit without the use of mechanical ventilation

Appendix 3 

**Table 5:** Details of AKI patients. AKI: acute kidney injury; CPB: cardiopulmonary bypass

Serum creatinine						Urine output (mL/kg/h)		Urine output criteria?	Fluid overload	CPB time
Case	Baseline	Day 1	Day 2	Day 3	Criterion 1	0 h	24 h	<0.5 mL/kg/h	>10%	
					>0.3 mg/dL 1.5-1.9 times baseline) raise					
1	33	52	48	43	yes	5	3.8	no	3.70%	72
2	33	62	40	42	yes	5.73	2.48	no	-0.60%	73
3	36	52	54	40	yes	2.4	4.5	no	-7.90%	83
4	38	59	39	33	yes	3.2	2.1	no	6.20%	90
5	37	54	83	57	yes	1.2	1.8	no	7.90%	95
6	41	60	63	40	yes	1.6	2.9	no	-3.50%	97
7	31	49	45	33	yes	4.5	5.9	no	-2.20%	104
8	31	52	39	37	yes	1.3	3.4	no	5%	104
9	33	52	39	37	yes	1.66	3.43	no	4.50%	104
10	37	56	43	37	yes	3.51	5.38	no	-8.60%	105
11	24	34	38	49	yes	5.6	2.7	no	-24.40%	109
12	34	47	64	60	yes	1.3	1.3	no	-1.30%	130
13	35	57	47	39	yes	0.8	2.5	no	6.20%	143
14	36	56	54	38	yes	2	2.3	no	4.40%	150
15	41	64	75	67	yes	4.42	2.13	no	-11%	150
16	33	49	52	37	yes	1.1	2.2	no	12%	155
17	31	48	46	40	yes	2.82	4.71	no	-14.20%	160
18	36	52	54	40	yes	4.67	3.3	no	-33.50%	171
19	32	49	51	43	yes	1.8	4.3	no	14.80%	186
20	25	48	84	108	yes	0.3	0.8	yes	36%	200
21	34	36	52	43	yes	0.8	2.5	no	6%	202
22	42	47	66	56	yes	1.3	2.2	no	-11.50%	206
23	36	54	42	38	yes	2.5	4.4	no	-6.50%	207
24	45	54	62	74	no	0.61	0.36	yes	6.30%	219
25	64	80	122	186	yes	2.26	0.91	no	16.80%	240
26	40	61	66	67	yes	3.37	0.63	no	-8%	255
27	38	52	55	57	yes	7.6	2	no	-22.90%	263
